# Effects of digitalized Tai Chi intervention on muscle function and physical performance in older adults: a systematic review and meta-analysis

**DOI:** 10.3389/fpubh.2026.1793728

**Published:** 2026-03-18

**Authors:** Fengmei Gu, Jian Zhang, Wenjia Chen

**Affiliations:** 1 Department of Physical Education, College of Physical Education, Chuzhou University, Chuzhou, China; 2 Department of Physical Education, School of Physical Education, China University of Mining and Technology, Xuzhou, China

**Keywords:** digitalized, meta-analysis, muscle function, older adults, physical performance, randomized controlled trials (RCTs), Tai Chi

## Abstract

**Background and objective:**

With the rapid development of digital health technology, remote Tai Chi intervention has gained significant attention as a novel model for geriatric rehabilitation. However, consistent evidence regarding the comprehensive effects of digitalized Tai Chi on muscle function and physical performance in older adults remains insufficient. This study aims to systematically evaluate the effects of digitalized Tai Chi intervention on muscle strength, physical function, and muscle mass in older adults.

**Methods:**

A systematic search was conducted across databases including PubMed, Web of Science, Cochrane Library, and CNKI to identify randomized controlled trials (RCTs) focusing on digitalized Tai Chi interventions for older adults. A random-effects model was employed to evaluate intervention effects, and sensitivity analysis was performed to explore sources of heterogeneity. The certainty of evidence for each outcome measure was assessed using the GRADE system.

**Results:**

Seven RCTs involving 469 participants were ultimately included. Meta-analysis showed that digitalized Tai Chi intervention had no statistically significant advantages over controls in handgrip strength [SMD = −0.12, 95% CI (−0.47, 0.23), *p* = 0.51], lower limb explosive power (30s-STS) [SMD = 0.24, 95% CI (−0.58, 1.06), *p* = 0.56], functional mobility (TUGT) [SMD = 0.17, 95% CI (−0.08, 0.42), *p* = 0.18], or walking endurance (6MWT) [SMD = −0.22, 95% CI (−0.60, 0.16), *p* = 0.26]. Although potential improvements in single-leg stance (SLS) were observed after excluding heterogeneous studies [SMD = 0.49, *p* = 0.03], and individual studies suggested benefits of 3D pose recognition for muscle mass (ASMI), these findings remain exploratory due to the single-trial evidence. The overall certainty of evidence was low or very low.

**Conclusion:**

Current evidence is insufficient to confirm that digitalized Tai Chi is superior to traditional rehabilitation or routine care in enhancing muscle strength and dynamic mobility in older adults. While high-precision digital feedback technology shows potential for improving static balance and muscle remodeling, its core efficacy remains highly uncertain. Future large-sample, high-quality RCTs focusing on closed-loop intensity monitoring and dynamic feedback mechanisms are required to further evaluate the clinical value of digitalized Tai Chi in geriatric rehabilitation.

**Systematic review registration:**

www.crd.york.ac.uk/prospero, identifier: CRD420261291107.

## 1 Introduction

With the acceleration of global population aging, the decline of muscle function in older adults (e.g., sarcopenia) and the resulting deterioration in physical performance have become severe challenges in the field of global public health (1, 2). Muscle function involves more than just the maintenance of muscle mass; more crucially, it is reflected in the output of muscle strength, serving as the physiological cornerstone for older adults to maintain their independent living capacity (3). Studies have shown that skeletal muscle loss and the functional decline of the neuromuscular system that occur with age directly lead to the worsening of physical performance, manifested as impaired balance, reduced gait speed, and decreased Timed Up and Go (TUG) ability (4, 5). This decline not only significantly increases the risks of falls, fractures, and disability (6), but is also closely associated with a decrease in the quality of life and a surge in medical burdens among older adults (7). Therefore, seeking safe and effective interventions to delay or reverse the degradation of muscle function and physical performance in older adults has become an urgent priority in geriatric medicine and sports science.

Among various non-pharmacological interventions, Tai Chi has been widely proven to have unique value in improving balance ability and enhancing core muscle strength in older adults due to its emphasis on weight shifting, lower limb stability, and movement coordination (8, 9). However, the traditional face-to-face teaching model faces barriers such as geographical limitations, uneven distribution of professional coaching resources, and a lack of real-time, objective corrective feedback during home practice (10, 11). In recent years, with the increasing maturity of various digital technologies, digitalized intervention methods have developed rapidly in the field of health promotion. Through the integration of technologies such as Artificial Intelligence (AI) pose estimation, wearable sensors, Virtual Reality (VR), and remote video platforms, Digitalized Tai Chi has emerged (12, 13). This novel model breaks through spatial and temporal constraints, achieving precision in exercise monitoring and real-time feedback, thereby enhancing the scientific rigor and adherence of practice while providing innovative digital solutions for geriatric health management (14, 15).

Current evidence-based medical evidence has preliminarily confirmed the positive benefits of digitalized mind-body exercise interventions. A systematic review by Li et al. (16) in 2025 showed that Tai Chi interventions based on Information and Communication Technology, or ICT, were significantly superior to traditional practice models in improving global cognitive function and physical mobility performance, such as the TUG, 30-second chair stand, and gait speed, in patients with mild cognitive impairment, highlighting the advantages of digital interaction in promoting rehabilitation. A meta-analysis by Tu et al. (17) on digitalized traditional fitness exercises also confirmed that such interventions have a clear promoting effect on improving mobility, balance function, and cognitive levels in older adults. However, while digitalized programs have shown positive effects on cognition and macro-mobility, existing research results still exhibit significant inconsistencies regarding core indicators reflecting muscle strength, such as handgrip strength and lower limb explosive power (18). First, the effect on strength improvement remains controversial; previous studies have found that digitalized exercises did not yield significant benefits in improving handgrip strength in older adults, suggesting that existing digitalized programs may have deficiencies in the design of strength reinforcement. Second, there is a lack of focus on muscle mass indicators; current reviews mostly focus on cognition, balance, or macro-mobility indicators, while the changes in the skeletal muscle mass index, a key indicator for preventing sarcopenia, during digitalized Tai Chi intervention remain in an evidence vacuum (19). Furthermore, due to the enormous heterogeneity in the technological pathways adopted across various studies, it has not yet been systematically elucidated how digitalized feedback mechanisms are precisely transformed into long-term adaptations of the neuromuscular system.

Based on these research gaps, this study aims to comprehensively and quantitatively evaluate the integrated effects of digitalized Tai Chi intervention on muscle mass, muscle strength, and physical performance in older adults through a systematic review and meta-analysis. By delving into the regulatory effects of different digitalized technological pathways on intervention outcomes, this research seeks to address the insufficient evidence-based support in the field of quantitative improvement of physiological functions through digitalized mind-body exercises. Ultimately, it aims to provide a more objective and systematic reference for formulating precise exercise prescriptions in clinical practice.

## 2 Materials and methods

The protocol for this study has been registered with PROSPERO (Registration No: CRD420261291107), which clearly defined the primary objectives, inclusion and exclusion criteria, interventions, control measures, and the planned primary and secondary outcome measures. The implementation of this systematic review strictly adhered to the pre-registered protocol without significant deviations. Furthermore, the study was conducted and reported in strict accordance with the Preferred Reporting Items for Systematic Reviews and Meta-Analyses (PRISMA 2020) checklist (20).

### 2.1 Search strategy

This study systematically searched five English databases, including PubMed, Web of Science, EMBASE, Cochrane Library, and CINAHL, as well as three Chinese databases, namely China National Knowledge Infrastructure (CNKI), VIP, and Wanfang. The search period spanned from the inception of each database until January 14, 2026, aimed at identifying randomized controlled trials (RCTs) investigating the impact of digitalized Tai Chi interventions on muscle function and physical performance in older adults. The search utilized a combination of Medical Subject Headings (MeSH) and free-text words. The search terms were constructed around research subjects such as older adults and advanced age; intervention programs like Tai Chi; technological pathways including digitalization, virtual reality (VR), augmented reality (AR), telerehabilitation, and wearable devices; outcome measures such as muscle function, physical performance, handgrip strength, Timed Up and Go (TUG) tests, 30 s chair stand tests, and balance assessments; and research designs like randomized controlled trials. Search strings were adjusted according to the indexing rules of different databases, with detailed search strategies provided in Supplementary material 1. Additionally, to minimize the risk of omitting relevant studies, the reference lists of included articles were manually searched to supplement potential eligible studies.

### 2.2 Inclusion and exclusion criteria

The PICOS framework was utilized to establish the inclusion criteria. Regarding participants, older adults aged 60 and above were included, comprising healthy community residents or individuals with age-related chronic diseases such as sarcopenia, cognitive impairment, and fall risk. Interventions were limited to Tai Chi programs based on digital technologies, such as virtual reality, augmented reality, smartphone applications, wearable sensor feedback, or real-time interactive teaching via video conferencing platforms. The control groups received non-digitalized interventions, such as traditional offline face-to-face Tai Chi instruction, other conventional exercises, health education, or a blank control maintaining daily lifestyle habits. Outcome measures were required to include objective measurements of muscle function or physical performance, such as the Timed Up and Go Test, 30 s chair stand test, handgrip strength, and balance assessments including the Berg Balance Scale and Single-Leg Stance (SLS) test. The study design was strictly limited to randomized controlled trials. Corresponding exclusion criteria included: experimental groups using traditional non-digital face-to-face models; non-randomized controlled trials such as single-arm studies or quasi-experimental designs; reviews, conference abstracts, letters, editorials, or case reports; and animal experiments or pilot studies with missing data or incomplete results reporting.

### 2.3 Data collection

Two researchers independently extracted data from the included studies and recorded the information using a standardized data extraction form. Extracted content included research identifiers such as the first author, publication year, and country; participant characteristics like age, sample size, and health status; intervention details such as the specific form of digitalized Tai Chi, intervention frequency, duration per session, and total duration; as well as control group types and core outcome measures including Timed Up and Go, 30 s chair stand, handgrip strength, and balance scores. For studies reporting multiple follow-up points, endpoint data at the completion of the intervention were extracted. Regarding data processing, if a study did not report the mean and standard deviation of the change from baseline, the mean change was calculated by subtracting the baseline mean from the post-intervention mean. The standard deviation of the change was estimated according to formulas recommended in the Cochrane Handbook. If continuous variables were reported as medians and interquartile ranges (IQR), we converted them to means and standard deviations using the method proposed by Wan et al. (21, 22). In cases of missing data, attempts were made to contact the corresponding authors. Any disagreements during the extraction process were resolved through discussion, with a third researcher making the final decision if necessary.

### 2.4 Risk of bias and certainty of evidence

The quality of the included studies was evaluated using the Cochrane Risk of Bias tool for randomized trials 2.0 (RoB 2) (23). The assessment covered five domains: bias arising from the randomization process, bias due to deviations from intended interventions, bias due to missing outcome data, bias in measurement of the outcome, and bias in selection of the reported result. Each domain was judged as “low risk,” “some concerns,” or “high risk,” leading to an overall risk of bias determination for each study. The certainty of evidence was evaluated using the GRADE system (24, 25), assessing for downgrading based on five aspects: risk of bias, inconsistency, indirectness, imprecision, and publication bias. The quality of evidence was categorized into four levels: high, moderate, low, and very low. Two evaluators independently completed the quality assessment, reaching consensus through discussion or by consulting a third expert if necessary.

### 2.5 Data analysis

The primary statistical analysis of this study was performed using Review Manager 5.4 software. Since the outcome measures—Timed Up and Go, handgrip strength, single-leg stance, 30 s chair stand, and 6 min walk test—were all continuous variables, the pooled effect sizes were estimated by calculating the standardized mean difference (SMD) and 95% confidence intervals (CI). Statistical heterogeneity was assessed using the I^2^ test, where I^2^ ≤ 25% indicated low heterogeneity, 25% to 50% indicated moderate heterogeneity, 50% to 75% indicated significant heterogeneity, and I^2^ ≥ 75% indicated high heterogeneity. Although models are typically selected based on the level of heterogeneity, a random-effects model was uniformly applied to the pooled analysis of all indicators in this study for conservatism and to improve the robustness of the conclusions. Sensitivity analysis was conducted by sequentially excluding individual studies to verify the stability of the pooled results. Exploratory subgroup analyses were performed to investigate potential sources of heterogeneity. Studies were categorized into high-interaction groups characterized by AI pose estimation, wearable sensor feedback, or VR with automated correction and real-time biofeedback, and low-interaction groups relying on manual feedback via online or tele-video platforms (26). Furthermore, subgroup analyses were conducted based on intervention durations, categorized as 12 weeks or less vs. more than 12 weeks (27). Publication bias was assessed using funnel plots and Egger's tests for quantitative evaluation when a single outcome measure included 10 or more studies; these analyses were not performed if fewer than 10 studies were included. All statistical significance levels were set at *p* < 0.05.

## 3 Results

### 3.1 Study selection

**Figure 1:**
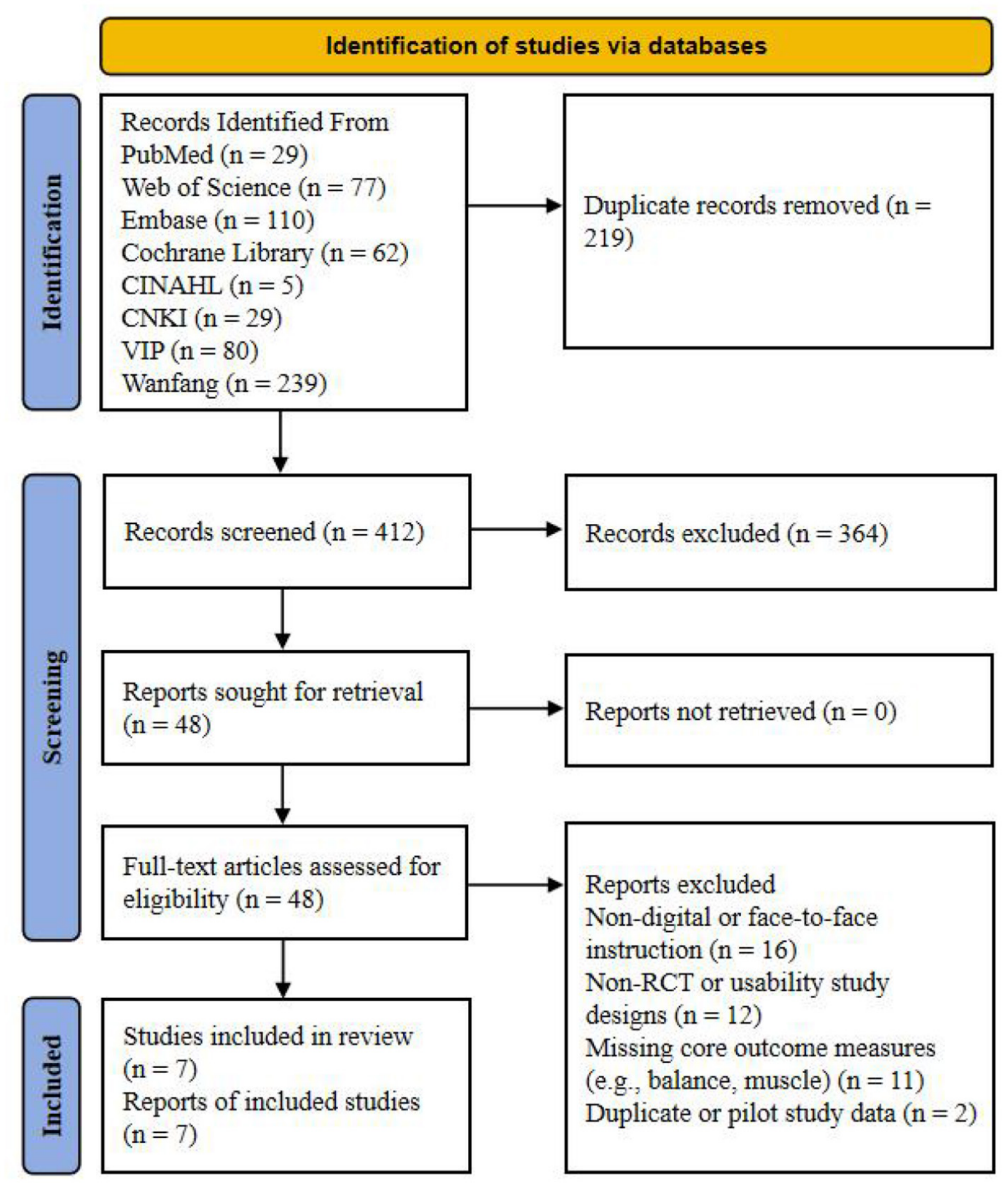
Preferred reporting items for systematic reviews and meta-analysis (PRISMA) study flow diagram.

A systematic study selection was conducted in accordance with the PRISMA 2020 guidelines, and the selection process is illustrated in Figure 1. Through a comprehensive search across eight databases, including PubMed, Web of Science, Cochrane Library, Embase, CINAHL, CNKI, VIP, and Wanfang, a total of 631 records were initially identified. After 219 duplicate records were removed via automatic and manual screening, the remaining 412 records underwent title and abstract screening, resulting in the exclusion of 364 documents that clearly did not meet the eligibility criteria. Subsequently, 48 potentially relevant reports were evaluated through full-text assessment, and 40 reports were excluded according to pre-specified criteria. Specifically, 16 reports were excluded for using non-digital interventions or offline face-to-face instruction models; 12 reports were excluded because they were non-randomized controlled trials or single-arm usability study designs; two reports were excluded due to being pilot studies or having data overlap; and 11 reports were excluded for lacking core physical performance or muscle-related indicators. Ultimately, seven randomized controlled trials were included in the quantitative synthesis and meta-analysis.

### 3.2 Study characteristics

Table 1 summarizes the primary characteristics of the seven included RCTs (28–34), detailing information such as the first author, country or region, publication year, participant characteristics, intervention protocols, and core outcome measures. These studies were published between 2010 and 2024 (28, 29), involving a total of 469 participants. Regarding geographical distribution, the majority of the studies were conducted in China (*n* = 5) (29, 30, 32–34), while the remaining were carried out in the United States (*n* = 2) (28, 31). The baseline characteristics of the participants encompassed various older adult populations, including community-dwelling healthy older adults (29, 32, 34), individuals with cognitive impairment, such as CI or MCI (30, 31), patients with sarcopenia (33), and groups at risk for falls (28). The sample sizes of the included studies ranged significantly, from 28 to 212 cases (31, 34), with the mean age of participants primarily concentrated between 60 and 80 years (29, 31).

**Table 1:** Characteristics of the included studies.

**References**	**Country/region**	**Sample size (*N*) (E/C)**	**Age (mean ±SD) (E/C)**	**Participant characteristics (P)**	**Intervention (I): digital means and content**	**Control (C): specific content**	**Period/frequency**	**Core outcomes (O)**
Chen et al. (5)	China	14/14	72.2 ± 7.8/75.1 ± 5.5	Community-dwelling healthy older adults	Augmented Reality (AR) assistance: Tai Chi training with real-time comparison between individual movements and a virtual coach via a display	Traditional on-site teaching: Standard face-to-face verbal instruction and demonstration by a coach	8 weeks, 3 sessions/week, 60 min	BBS, TUG, 30s-STS
He et al. (33)	China	24/23	73.67 ± 4.77/70.91 ± 3.94	Older adults aged 60 to 75 with sarcopenia	3D pose estimation telerehabilitation: Remote Tai Chi practice monitored and evaluated using deep learning pose estimation technology	Traditional face-to-face training: Standard offline demonstration and instructional teaching provided by a coach	12 weeks, 3 sessions/week, 40 min	ASMI, Handgrip strength, TUGT, Gait speed
Hsieh et al. (30)	China	31/29	76.4 ± 7.6/80.0 ± 7.5	Older adults over 65 with cognitive impairment (CI)	Virtual Reality (VR) interaction: Modified Tai Chi practice using Xbox 360 Kinect with movement sensor feedback	Routine life control: Maintaining original daily activities without additional exercise intervention	24 weeks, 2 sessions/week, 60 min	TUGT, 30s-STS, Gait speed
Li et al. (31)	USA	105/107	76.0 ± 5.1/75.9 ± 5.1	Middle-aged and older adults with MCI or self-reported memory concerns	Cognitively enhanced online video: Real-time guidance via Zoom, integrating cognitive challenge tasks into Tai Chi	Standard online Tai Chi: Standard Tai Chi training conducted via Zoom video conferencing	24 weeks, 2 sessions/week, 60 min	TUG, 30s-STS
Li et al. (29)	China	21/29	65.2 ± 4.2/64.9 ± 3.7	Healthy older men aged 60 to 70	IMU+AI precise movement feedback: Real-time movement recognition and corrective feedback using wearable sensors and neural networks	Standard intervention group: Standard Tai Chi practice without sensor monitoring or AI-based personalized feedback	8 weeks, 3 sessions/week, 40 min	Handgrip strength, Single-leg stance balance
Wu et al. (28)	USA	22/20	76.1 ± 7.9/74.1 ± 6.9	Community-dwelling older adults with fall risk or fear of falling	Remote real-time video interaction: Real-time interactive practice of 24-form Tai Chi with a coach via the DocBox system on a television	Community traditional training: Face-to-face on-site teaching at a community center conducted by the same coach	15 weeks, 3 sessions/week, 60 min	TUG, SLS
Zhang et al. (32)	China	14/16	65.3 ± 5.1/65.7 ± 4.6	Community-dwelling older adults (Bafa Wubu practitioners) aged 60 to 70	Wearable AI feedback: Real-time movement correction and accuracy scoring via IMU sensors and a mobile App	Traditional verbal feedback: Manual observation and traditional oral correction suggestions provided by a coach	8 weeks, 3 sessions/week, 40 min	Handgrip strength, Balance control

*N* represents sample size; Mean ± SD represents mean and standard deviation; P represents participant characteristics; I represents intervention measures for the experimental group; C represents intervention measures for the control group; O represents core outcome measures. The definitions of abbreviations and measurement units are as follows: BBS refers to the Berg Balance Scale measured in points; TUG and TUGT both refer to the Timed Up and Go Test measured in seconds; 30s-STS refers to the 30 s chair stand test measured in repetitions; ASMI refers to the appendicular skeletal muscle mass index measured in kg/m^2^; SLS refers to the single-leg stance test measured in seconds; 6-MWT refers to the 6 min walk test measured in meters; and handgrip strength is measured in kilograms. MCI denotes mild cognitive impairment; CI denotes cognitive impairment; VR denotes virtual reality; AR denotes augmented reality; IMU denotes inertial measurement unit; and AI denotes artificial intelligence.

Regarding intervention measures, digitalized Tai Chi presented in various forms, including Augmented Reality (AR)-assisted training (34), remote monitoring based on 3D pose estimation (33), Virtual Reality (VR) interaction (30), real-time online video guidance, and motion feedback systems utilizing Inertial Measurement Units (IMU) and Artificial Intelligence (AI) (28, 29, 31, 32). This study further conceptualized these interventions into three dimensions. First, in terms of Feedback Modality, He et al. (33) and Li et al. (29) employed real-time biofeedback based on AI pose recognition or sensors, whereas Wu et al. (28) primarily relied on manual real-time feedback via video media. Second, regarding Automation Level, studies utilizing 3D vision and neural network algorithms demonstrated high-automation correction features, while protocols based on online video conferencing (e.g., Zoom) focused more on remote human interaction. Finally, in the Intensity Monitoring dimension, the core logic of the vast majority of current digital schemes [e.g., Hsieh et al. (30)] remains focused on precise posture matching; there is a universal lack of closed-loop monitoring and dynamic adjustment mechanisms for exercise load indicators, such as heart rate and Rating of Perceived Exertion (RPE), in older participants. Intervention durations ranged from 8 to 24 weeks (30, 34), with a frequency of 2 to 3 sessions per week (30, 34), lasting 40 to 60 min per session (28, 33). Control groups primarily received traditional in-person guidance (28, 33), standardized online Tai Chi training, or no additional intervention (blank control) (29–32, 34). Regarding outcome measures, most studies utilized the TUGT and 30s-STS to evaluate physical performance, with handgrip strength and various balance scales used as indicators for muscle function and postural control.

### 3.3 Risk of bias

**Figure 2:**
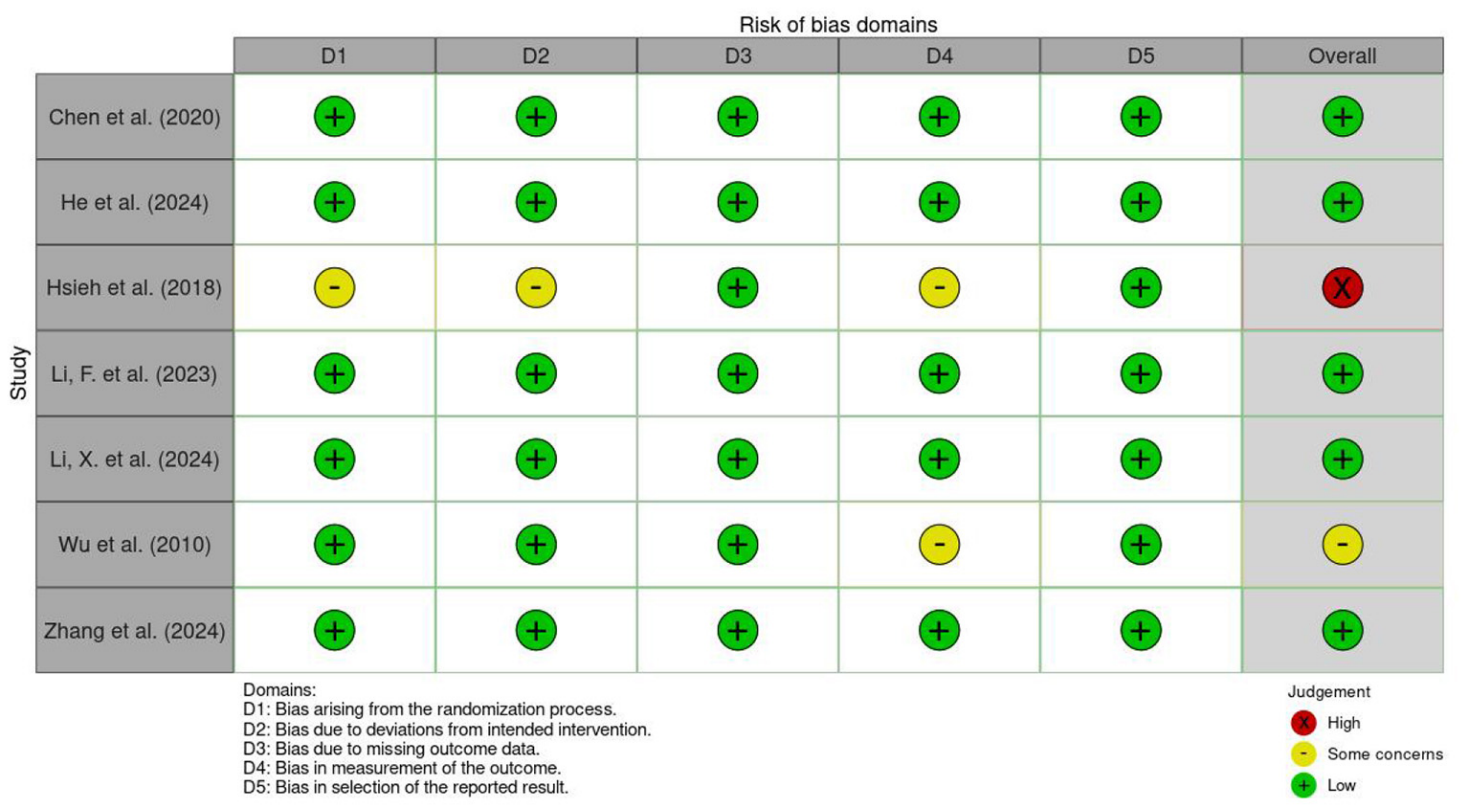
Risk of bias summary: review of the authors judgments about each risk of bias item for each included study.

**Figure 3:**
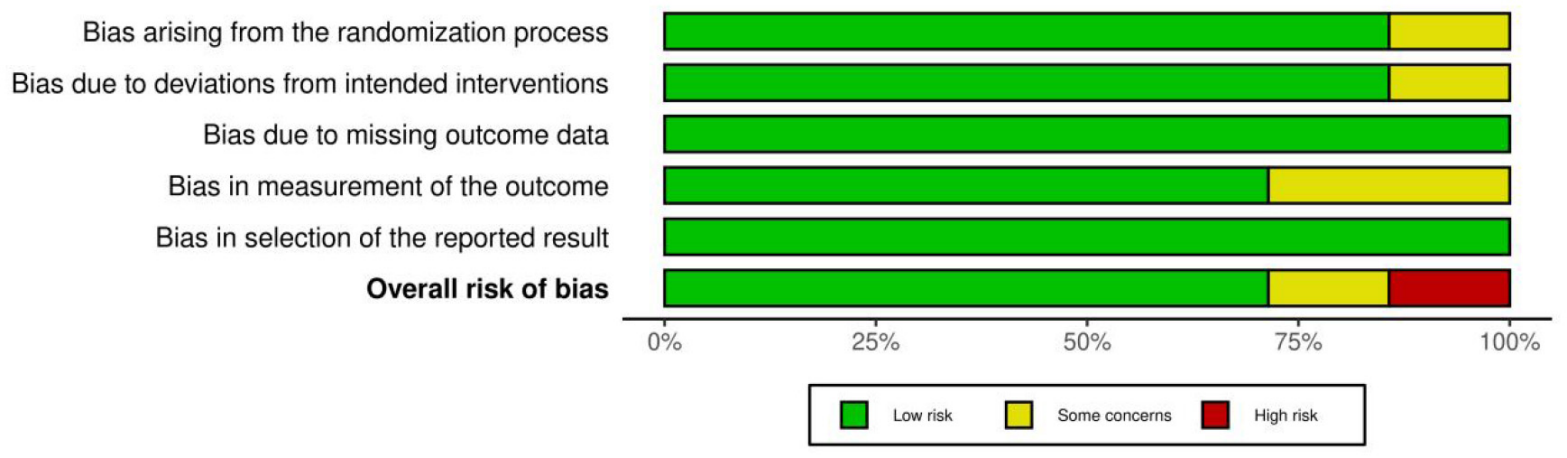
Risk of bias graph: review authors' judgments about each risk of bias item, presented as percentage of included studies.

In this study, the quality of the seven included studies was evaluated using the Revised Cochrane Risk of Bias Tool for Randomized Trials (RoB 2.0), as shown in Figures 2, 3. Regarding the domain of the randomization process, the vast majority of the studies were judged to be at low risk due to detailed descriptions of random sequence generation and allocation concealment; only Hsieh et al. (30) was judged to have some concerns because the descriptions of sequence generation and allocation concealment were insufficiently detailed. In the domain of deviations from intended interventions, most studies exhibited a low risk of bias, while only Hsieh et al. (30) was judged to have some concerns due to its data analysis strategy and potential influences during the intervention implementation process. In the domain of missing outcome data, all included studies demonstrated high data integrity and were judged to be at low risk overall. In the domain of outcome measurement, most studies complied with assessment standards, whereas Wu et al. (28) and Hsieh et al. (30) were judged to have some concerns due to inadequate explanations regarding the implementation and maintenance of blinding. In the domain of selective reporting of results, all studies showed high reporting transparency and were all judged to be at low risk. Integrating the assessment results across the five domains, this study ultimately determined that five studies were at low risk of bias, while Wu et al. (28) was judged to have some concerns, and Hsieh et al. (30) was judged to be at high risk due to cumulative bias across multiple domains.

### 3.4 Meta-analysis results

#### 3.4.1 Muscle strength

**Figure 4:**
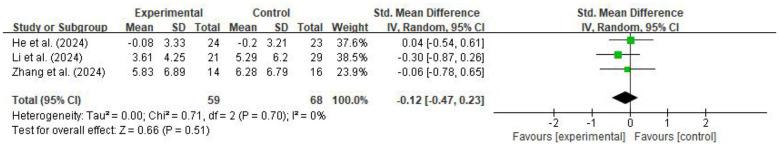
Forest plot of the effect of digitalized Tai Chi intervention on handgrip strength in older adults.

A total of three studies evaluated the effect of digitalized Tai Chi intervention on handgrip strength in older adults, involving 59 participants in the experimental group and 68 in the control group, as shown in Figure 4. The pooled analysis using a random-effects model indicated that there was no statistically significant difference between the digitalized Tai Chi group and the control group regarding handgrip strength improvement [SMD = −0.12, 95% CI (−0.47, 0.23), *Z* = 0.66, *p* = 0.51]. The heterogeneity test showed high homogeneity across the studies (I^2^ = 0%, *p* = 0.70). These results suggest that, within the sample scope of this study, the effect of digitalized Tai Chi intervention on enhancing upper limb muscle strength in older adults is not significant.

**Figure 5:**
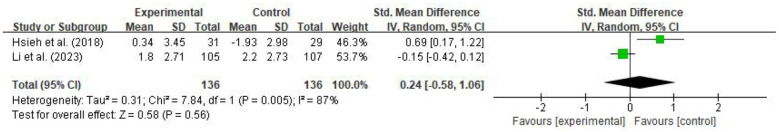
Forest plot of the effect of digitalized Tai Chi intervention on 30s-STS in older adults.

Two studies assessed the impact of digitalized Tai Chi intervention on lower limb strength (30s-STS) in older adults, involving a total of 272 participants, with 136 in the experimental group and 136 in the control group, as shown in Figure 5. Pooled analysis using a random-effects model showed that the difference in 30s-STS performance between the digitalized Tai Chi group and the control group was not statistically significant [SMD = 0.24, 95% CI (−0.58, 1.06), *Z* = 0.58, *p* = 0.56]. Heterogeneity testing revealed significant heterogeneity between studies (I^2^ = 87%, *p* = 0.005). The pooled results indicate that current data do not demonstrate a significant advantage for digitalized Tai Chi intervention in improving lower limb muscle strength in older adults compared to control groups.

#### 3.4.2 Physical performance

**Figure 6:**
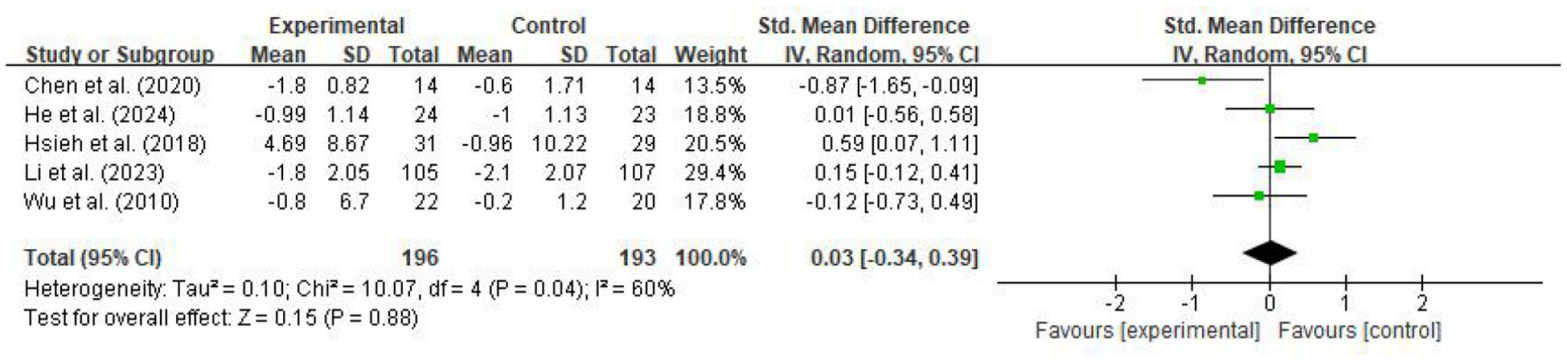
Forest plot of the effect of digitalized Tai Chi intervention on TUGT in older adults.

The meta-analysis for the Timed Up and Go Test (TUGT) initially included five studies and showed a moderate degree of heterogeneity (SMD = 0.03, 95% CI: −0.34 to 0.39, *p* = 0.88; I^2^ = 60%), with the pooled effect size showing no statistical significance, as shown in Figure 6. Sensitivity analysis conducted by sequentially excluding individual studies identified Chen et al. (34) as a significant source of outliers. After excluding this study, the model heterogeneity decreased substantially from 60% to 20%, reaching a level of low heterogeneity. Although the pooled result after exclusion showed a slight numerical shift (SMD = 0.17, 95% CI: −0.08 to 0.42, *p* = 0.18), it did not reach statistical significance. This indicates that the impact of digitalized Tai Chi intervention on TUGT in older adults is relatively robust; namely, current evidence has not confirmed that this intervention model is superior to control groups in reducing TUGT time.

**Figure 7:**
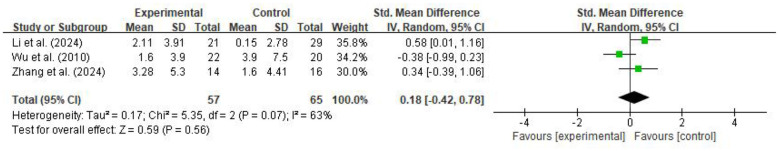
Forest plot of the effect of digitalized Tai Chi intervention on SLS in older adults.

The meta-analysis for the single-leg stance (SLS) test initially included three studies involving 122 participants, as shown in Figure 7. Initial pooled results indicated that the improvement in SLS performance following digitalized Tai Chi intervention was not statistically significant [SMD = 0.18, 95% CI (−0.42, 0.78), *p* = 0.56], with significant heterogeneity observed among studies (I^2^ = 63%). Sensitivity analysis through sequential exclusion revealed that Wu et al. (28) was the primary source of heterogeneity, as its result (SMD = −0.38) deviated markedly from the overall positive trend. After excluding this study, the model heterogeneity dropped to 0%, and the pooled effect size shifted to a statistically significant positive result [SMD = 0.49, 95% CI (0.04, 0.94), *p* = 0.03].

**Figure 8:**
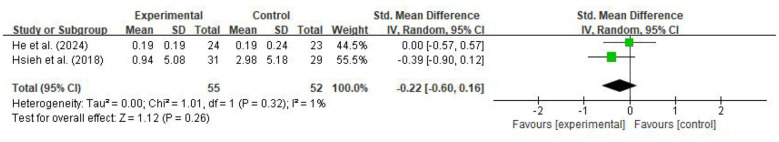
Forest plot of the effect of digitalized Tai Chi intervention on 6MWT in older adults.

Two studies evaluated the impact of digitalized Tai Chi intervention on the 6 min walk test (6MWT) in older adults, involving 107 participants, with 55 in the experimental group and 52 in the control group, as shown in Figure 8. Pooled analysis using a random-effects model showed that the difference in 6MWT performance between the digitalized Tai Chi group and the control group was not statistically significant [SMD = −0.22, 95% CI (−0.60, 0.16), *Z* = 1.12, *p* = 0.26]. Heterogeneity testing showed high homogeneity across studies (I^2^ = 1%, *p* = 0.32). These findings suggest that, within the current sample, data do not observe a significant advantage for digitalized Tai Chi intervention in improving long-distance walking endurance in older adults compared to control groups.

#### 3.4.3 Muscle mass

Since most studies included in this review did not report data related to muscle mass, a meta-analysis could not be performed. Instead, a systematic descriptive analysis was conducted on the study by He et al. (33), which reported this outcome. This study implemented a digitalized Tai Chi tele-intervention based on 3D human pose estimation technology for older adults with sarcopenia. Results showed that after completing 12 weeks of Tai Chi practice at a frequency of three sessions per week, the ASMI of participants in the AI-based tele-training group significantly increased from 4.79 ± 0.79 kg/m^2^ at baseline to 5.16 ± 0.80 kg/m^2^ (*p* < 0.01). Given that the evidence stems from only a single RCT, these findings should be regarded as exploratory and hypothesis-generating preliminary results rather than confirmatory evidence; their robustness remains to be validated by future research.

### 3.5 Exploratory subgroup analysis

Exploratory subgroup analyses were performed for the TUGT based on the level of technological interaction and the intervention duration. The results are summarized in Table 2, with specific subgroup forest plots provided in Supplementary Figures 1, 2. The subgroup analysis for technological interaction level included three studies in the high-interaction group, where the pooled effect size showed no statistical significance [SMD = −0.04, 95% CI (−0.81, 0.73), *p* = 0.91] alongside high within-group heterogeneity. The low-interaction group included two studies, which also showed no significant difference [SMD = 0.10, 95% CI (−0.14, 0.35), *p* = 0.42] but exhibited high within-group homogeneity. The test for subgroup differences yielded *p* = 0.73, indicating that the level of technological interaction did not exert a significant moderating effect on the intervention outcomes (33). Regarding intervention duration, the short-term intervention group included two studies, with pooled results showing no statistical significance [SMD = −0.39, 95% CI (−1.24, 0.47), *p* = 0.37] and significant within-group heterogeneity. The medium-to-long-term intervention group included three studies, where the pooled effect size similarly failed to reach statistical significance [SMD = 0.21, 95% CI (−0.13, 0.54), *p* = 0.22] despite low within-group heterogeneity. The test for subgroup differences yielded *p* = 0.20, suggesting that the duration of the intervention did not significantly moderate the intervention effect. The overall pooled results indicated that the difference between the digitalized Tai Chi intervention and the control group in improving TUGT performance in older adults was not statistically significant [SMD = 0.03, 95% CI (−0.34, 0.39), *p* = 0.88, I^2^ = 60%].

**Table 2:** Summary of exploratory subgroup analysis results for the effect of digitalized Tai Chi intervention on TUGT performance in older adults.

**Subgroup category**	**Subgroup level**	**Number of studies**	**Sample size (experimental/control)**	**SMD (95% CI)**	**Within-group *p*-value**	**I^2^ (%)**	***P*-value for subgroup differences**
Technological interaction level	High-interaction group	3	69/66	−0.04 (−0.81, 0.73)	0.91	79%	0.73
	Low-interaction group	2	127/127	0.10 (−0.14, 0.35)	0.42	0%	
Intervention duration	≤ 12 weeks	2	38/37	−0.39 (−1.24, 0.47)	0.37	68%	0.2
	> 12 weeks	3	158/156	0.21 (−0.13, 0.54)	0.22	41%	
Overall pooled		5	196/193	0.03 (−0.34, 0.39)	0.88	60%	

### 3.6 Publication bias

The number of original studies included for all core outcome measures in this study, including the Timed Up and Go Test, single-leg stance test, handgrip strength, 30 s chair stand test, and 6 min walk test, was less than 10. This did not meet the minimum methodological requirements for conducting quantitative assessments of publication bias, such as funnel plot analysis or Egger's test. Consequently, no statistical tests for publication bias were performed for the outcome measures in this research. The pooled analysis results for these indicators should be interpreted with caution, considering their respective sample sizes and certainty of evidence.

### 3.7 Certainty of evidence

This study utilized the GRADE (Grading of Recommendations Assessment, Development and Evaluation) system to assess the certainty of evidence for the primary outcome measures, as summarized in Table 3. Influenced by factors such as risk of bias, inconsistency, and imprecision, the overall certainty of evidence regarding the effects of digitalized Tai Chi intervention on physical function in older adults remained at a low level. Specifically, the certainty of evidence for the Timed Up and Go Test, 30 s chair stand test, and 6 min walk test was rated as “very low.” This was primarily attributed to the high risk of bias in some studies and the significant statistical heterogeneity observed in the outcome measures. Furthermore, the limited number of included studies resulted in wide confidence intervals for the pooled effect sizes, all of which crossed the line of no effect. The certainty of evidence for the single-leg stance test and handgrip strength was rated as “low.” Although the studies on handgrip strength exhibited high homogeneity, the evidence level was downgraded due to the limited total number of participants and the high imprecision of the pooled results. Additionally, the lack of quantitative assessment for publication bias, resulting from having fewer than 10 studies for each indicator, further restricted the certainty of the evidence.

**Table 3:** Certainty of the evidence using the GRADE approach for main outcomes.

**Outcomes**	**Participants (studies)**	**Risk of bias**	**Inconsistency**	**Indirectness**	**Imprecision**	**Publication bias**	**Pooled effect size (95% CI)**	**Certainty of evidence**
TUGT	389 (5)	Serious (a)	Serious (b)	Not serious	Serious (c)	Not evaluated (d)	SMD = 0.03 [−0.34, 0.39]	⊕○○○ Very low
SLS	122 (3)	Serious (a)	Serious (b)	Not serious	Serious (c)	Not evaluated (d)	SMD = 0.18 [−0.42, 0.78]	⊕⊕○○ Low
Grip strength	127 (3)	Serious (a)	Not serious	Not serious	Serious (c)	Not evaluated (d)	SMD = −0.12 [−0.47, 0.23]	⊕⊕○○ Low
30s-STS	272 (2)	Serious (a)	Very serious (b)	Not serious	Very serious (c)	Not evaluated (d)	SMD = 0.24 [−0.58, 1.06]	⊕○○○ Very low
6-MWT	107 (2)	Serious (a)	Not serious (e)	Not serious	Very serious (c)	Not evaluated (d)	SMD = −0.22 [−0.60, 0.16]	⊕○○○ Very low

(a) Some studies had deficiencies in the description of the randomization process or failed to implement blinding; (b) Significant statistical heterogeneity was observed (I^2^ > 50%); (c) The sample size was small, and the confidence intervals crossed the line of no effect; (d) The number of included studies was fewer than 10, and no quantitative assessment of publication bias was performed; (e) High homogeneity was observed between studies (I^2^ = 1%).

## 4 Discussion and analysis

### 4.1 Summary of findings

The analysis of data from 469 participants across seven randomized controlled trials in this study indicates that the overall effect of digitalized Tai Chi intervention on improving muscle function and physical performance in older adults currently remains in an exploratory stage. The results show that the digitalized intervention group has not yet demonstrated a statistically significant advantage over control groups in terms of enhancing upper limb muscle strength, such as handgrip strength, or long-distance walking endurance, such as the 6 min walk test; furthermore, relevant indicators exhibited high consistency across the included studies. Regarding the 30 s chair stand test and the Timed Up and Go Test, which reflect lower limb explosive power and functional mobility, although some studies showed a positive improvement trend, the overall pooled effect size did not reach a level of significance and was accompanied by moderate to high statistical heterogeneity. This suggests that, compared to traditional exercises or baseline controls, the efficacy of existing digitalized intervention models in inducing substantial improvements in macro-physical functions may be constrained by the precision of the intervention methods and the stimulus intensity of the exercise load.

Although potential positive signals in balance improvement (e.g., Single-Leg Stance test) were observed through sensitivity analysis, the robustness of this conclusion remains to be further verified in the future. Notably, regarding the core indicator of muscle mass, the descriptive analysis of this study shows that schemes adopting high-precision feedback technologies such as 3D human pose estimation demonstrate potential prospects for improvement. However, it must be clear that this finding currently only holds hypothesis-generating value. Due to the lack of pooled validation from multiple studies, confirmatory conclusions cannot yet be drawn based on this. Furthermore, exploratory subgroup analysis initially shows that the interactive depth of digital technology and the intervention duration have not yet produced significant moderating effects on core physical performance indicators. Given that the evidence quality of the currently included outcome indicators is mostly at low or very low levels in the GRADE system, and the amount of primary literature is limited, the findings of this study should be regarded as preliminary speculative conclusions. In future clinical promotion, although digitalized Tai Chi possesses the potential convenience of remote health promotion, its core efficacy in strengthening muscle function still requires more high-quality, large-sample studies for cautious evaluation.

### 4.2 Theoretical speculations and analysis of factors influencing the impact of digitalized Tai Chi on muscle function and physical performance in older adults

Regarding the phenomenon where no significant improvements were observed in muscle function indicators, this study proposes the following speculations from a theoretical perspective. First, this may stem from the non-specific matching between participant characteristics and the logic of digital interventions. Although a large number of randomized controlled trials (RCTs) have confirmed that traditional Tai Chi yields clear benefits for physical function in older adults (35, 36), the RCT subjects included in this study spanned a wide range, covering healthy older adults, patients with cognitive impairment, and sarcopenic populations. This population heterogeneity may have led to a severe dilution of effect sizes. For instance, research by Hsieh et al. suggests that when faced with virtual interactions, older adults with cognitive impairment may need to allocate substantial executive functional resources to decode digital instructions. This cognitive load might interfere with their focus on movement quality and deep muscle engagement, resulting in neuromuscular recruitment effects that are inferior to those of healthy groups (30). Contrary to some studies claiming significant advantages of digital mind-body exercise in improving physical function (16, 17), this study failed to reach consistent positive conclusions on core indicators such as handgrip strength and the Timed Up and Go Test (TUGT). Such discrepancies may suggest that while digital interaction has potential in promoting rehabilitation engagement, its efficacy in achieving substantive breakthroughs in core physiological functions is constrained by the compatibility between the participants' baseline functional levels and digital interaction modes (37). In comparison, the study by Li et al. (29) on healthy older adults made breakthroughs in the balance dimension, yet handgrip strength indicators remained negative. This implies that even in cognitively healthy populations, if digital protocols lack personalized loading algorithms targeting specific physiological degeneration pathways such as neuromuscular conduction, the intervention efficacy may remain at the visual imitation stage, making it difficult to reach the deep requirements of functional remodeling (38).

Further analysis of the sources of heterogeneity revealed that the heterogeneity for the 30 s chair stand test, which reflects lower limb explosive power, was as high as 87%. This is not only related to differences in control group settings, such as blank controls vs. standard online training, but also reflects the instability of power transmission efficiency in digitalized interventions. Although potential positive signals were observed in the single-leg stance test in this study, reflecting the potential of digitalized interventions to improve static balance, a clear effect gap appeared when translating this into mobility that requires high-intensity muscle load and complex spatio-temporal coordination, such as the Timed Up and Go Test (39). This discrepancy in results may stem from the limitations of feedback mechanisms. Research by Zhang et al. (32) and Li X. et al. (29) in 2024 confirmed that sensors or artificial intelligence feedback can significantly improve balance performance by accurately identifying static center-of-gravity shifts. However, in dynamic tasks such as the Timed Up and Go Test involving multi-axial rotation and variable-paced movement, existing visual guidance still finds it difficult to replace the real-time sense of gravity resistance and explosive power guidance provided by physical coaches in person (34). Notably, He et al. (33) in 2024 found that the application of 3D pose estimation technology can significantly improve the Appendicular Skeletal Muscle Mass Index in sarcopenic older adults. This speculative finding suggests that ensuring the accuracy of posture through high-precision feedback helps induce ideal contraction and mechanical load in target muscle groups, thereby producing positive biological adaptations at the muscle mass level (40). However, when translating this into macro-physical performance, it is still necessary to overcome the limitations of digitalized feedback in enhancing motor control capabilities in dynamic, non-steady-state environments.

Additionally, a monitoring vacuum regarding exercise intensity in digital environments is also a key speculative factor for negative results (41). In traditional Tai Chi teaching, the on-site supervision of coaches and the group practice model can induce higher levels of psychological arousal and exercise intensity (42). In contrast, in most digitalized programs involved in this study, participants were mostly in a home-based practice state, such as the remote Zoom guidance model used by Li et al. (31). Due to the lack of real-time, closed-loop monitoring of heart rate, subjective perceived exertion, or muscle load intensity during the practice process, older adult participants may have unconsciously chosen safe but inefficient movement amplitudes. This results in intervention stimuli that have not yet reached the biological threshold required to induce significant growth in core functions (43). Combined with the results of the exploratory subgroup analysis in this study, the non-significant moderating effects (*p* > 0.05) of technological interaction levels and intervention duration further suggest that simply increasing technological complexity or extending practice time may be difficult for achieving a comprehensive leap in the physical performance of older adults if the core issues of precise delivery of effective exercise load and the lack of dynamic feedback are not resolved. This divergence of high technology and low efficacy suggests that future research and development of digitalized Tai Chi should shift from simple posture matching to a comprehensive intervention system that includes intensity regulation and dynamic proprioceptive reinforcement.

### 4.3 Research limitations

Although this study quantitatively evaluated the intervention effects of digitalized Tai Chi on muscle function and physical performance in older adults through systematic review and meta-analysis, several limitations remain due to the current state of primary research and objective constraints. First, the number of randomized controlled trials and the total sample size included in this study are limited. Ultimately, only 7 studies with a total of 469 participants met the inclusion criteria, which to some extent weakened the power of statistical inference. This made it difficult for some outcome indicators to cross the threshold of statistical significance, despite showing potential trends of change. Furthermore, because the number of publications for each outcome indicator was less than 10, this study could not conduct funnel plot analysis or Egger's test to quantitatively assess publication bias. Despite implementing an extensive database search, the potential impact of unpublished negative results on the pooled effect sizes cannot be entirely ruled out.

Second, significant heterogeneity exists in the participant populations and intervention pathways. The populations ranged from healthy older adults to various groups with cognitive impairment, sarcopenia, and fall risks. It must be clarified that due to this high level of population heterogeneity, the results of this study may not be directly generalizable to frail or highly clinical specific older adult populations. Meanwhile, the inconsistency in interactive depth and feedback precision among intervention protocols limited the ability of this study to provide precise recommendations.

Finally, the certainty level of evidence is limited and long-term evaluation data are lacking. According to the Grading of Recommendations Assessment, Development and Evaluation [GRADE] results, the quality of evidence for most core outcome indicators is only at low or very low levels. This is primarily related to the unclear description of randomization processes, the lack of blinding, and the imprecision caused by small sample sizes in the primary studies. Regarding the duration of intervention, existing research cycles are mostly concentrated between 8 and 24 weeks. For physiological indicators that require long-term load accumulation to produce substantive changes, such as skeletal muscle mass, the current observation periods may not yet have reached the threshold for inducing significant biological adaptation. Additionally, due to the lack of long-term follow-up data after the cessation of intervention, the sustained effects and safety of digitalized Tai Chi in improving muscle function in older adults still require further verification by future high-quality research.

### 4.4 Practical significance and implications

Although the findings of this study remain preliminary, the analysis provides a valuable reference for older adult health management and digital rehabilitation practices. Regarding the population focused on in this study, clinical application should remain cautious because the effects of digitalized Tai Chi on improving macro-performance including handgrip strength, TUGT, and walking endurance have not yet been robustly confirmed. It must be emphasized that when aiming for improvements in muscle strength or functional mobility, digital means should not currently be regarded as a substitute for traditional in-person supervised training; rather, they should serve as a supplementary strategy in environments where traditional rehabilitation resources are scarce. A hybrid model is recommended, providing necessary face-to-face guidance during the initial stage of intervention to ensure movement accuracy.

From policy and research perspectives, the results of this study support further exploration of the possibility of incorporating digital health technology into the prevention and control systems for chronic diseases in older adults. Policymakers should consider establishing standardized guidelines for digital mind-body exercise interventions and, while investing in digital infrastructure, strengthen the training of relevant skills for healthcare personnel. Given the current situation of low certainty of evidence and limited data, future research should focus on filling existing gaps, including conducting more large-sample, multi-center randomized controlled trials covering populations with different health baselines. Meanwhile, head-to-head comparative studies between different digital technology pathways should be strengthened. The minimum effective dose and potential mechanisms of digitalized Tai Chi intervention should be further clarified through the introduction of biomarkers, long-term follow-up monitoring, and the widespread adoption of key indicators such as Appendicular Skeletal Muscle Mass Index. Due to the low certainty of evidence and high heterogeneity of the participants, research conclusions should not be excessively extrapolated to frail or clinically severe populations. Furthermore, future technical research and development should attempt to transcend existing posture-matching frameworks and focus on more highly intelligent integrated systems. First, the application of wearable devices should be deepened, extending from basic exercise monitoring to the collection of multi-dimensional data on gait and balance. Second, these data should be used to develop predictive algorithms for the active identification and prevention of fall risks in older adults. Finally, the combination of AI virtual coaches with Augmented Reality (AR) or Virtual Reality (VR) technologies should be explored to dynamically adjust training content based on the real-time physiological feedback of participants, thereby constructing a highly personalized and immersive intelligent health management scheme for older adults.

## 5 Conclusion

Through a systematic review and meta-analysis of existing randomized controlled trials, this study found that digitalized Tai Chi intervention did not demonstrate statistically significant advantages over control groups in improving core functional indicators in older adults, such as handgrip strength, lower limb explosive power, dynamic mobility, and long-distance walking endurance. Although a potential positive trend was observed in the improvement of static balance ability, and individual studies employing high-precision 3D pose recognition technology showed preliminary exploratory signals in enhancing Appendicular Skeletal Muscle Mass Index, these findings remain highly uncertain due to the overall low certainty of evidence and the limited scale of the included literature. In summary, current evidence is insufficient to confirm that digitalized Tai Chi models are superior to traditional intervention models in strengthening muscle function in older adults. Especially when targeting improvements in strength and mobility, current digital means should not be regarded as a complete substitute for traditional offline face-to-face supervised training, and relevant conclusions should not be directly extrapolated to frail or highly clinical older adult populations. In future clinical practice, digitalized Tai Chi should be cautiously positioned as a supplementary tool for traditional rehabilitation. More large-sample studies are urgently needed to further verify its feasibility and effectiveness in the field of older adult rehabilitation by introducing closed-loop intensity monitoring and dynamic feedback systems.

## Data Availability

The datasets supporting the conclusions of this meta-analysis are included in the article and its Supplementary material. Search strategies and data extraction forms are available from the corresponding author upon reasonable request.

## References

[1] Beard JR Officer AM Cassels AK. The World Report on ageing and health. Gerontologist. (2016) 56:S163–6. doi: 10.1093/geront/gnw037 26994257

[2] Amuthavalli Thiyagarajan J Mikton C Harwood RH Gichu M Gaigbe-Togbe V Jhamba T . The UN Decade of Healthy Ageing: strengthening measurement for monitoring health and wellbeing of older people. Age Ageing. (2022) 51:afac147. doi: 10.1093/ageing/afac147 35776669 PMC9249069

[3] Bull FC Al-Ansari SS Biddle S Borodulin K Buman MP Cardon G . World Health Organization 2020 guidelines on physical activity and sedentary behaviour. Br J Sports Med. (2020) 54:1451–62. doi: 10.1136/bjsports-2020-102955 33239350 PMC7719906

[4] Cruz-Jentoft AJ Bahat G Bauer J Boirie Y Bruyère O Cederholm T . Sarcopenia: revised European consensus on definition and diagnosis. Age Ageing. (2019) 48:601. doi: 10.1093/ageing/afz046 31081853 PMC6593317

[5] Chen LK Woo J Assantachai P Auyeung TW Chou MY Iijima K . Asian Working Group for Sarcopenia: 2019 consensus update on sarcopenia diagnosis and treatment. J Am Med Dir Assoc. (2020) 21:300–7.e2. doi: 10.1016/j.jamda.2019.12.012 32033882

[6] Traverso A Bayram A Rossettini G Chiappinotto S Galazzi A Palese A. Investigating the biomechanics of falls in older adults in long-term care using a video camera: a scoping review. BMC Geriatr. (2024) 24:810. doi: 10.1186/s12877-024-05395-2 39367304 PMC11451165

[7] Esmaeilpour F Letafatkar A Karimi MT Khaleghi M Rossettini G Villafañe JH. Comparative analysis of ground reaction forces and spatiotemporal gait parameters in older adults with sway-back posture and chronic low back pain: a cross-sectional study. BMC Sports Sci Med Rehabil. (2025) 17:71. doi: 10.1186/s13102-025-01126-1 40197475 PMC11974159

[8] Huang D Ke X Jiang C Song W Feng J Zhou H . Effects of 12 weeks of Tai Chi on neuromuscular responses and postural control in elderly patients with sarcopenia: a randomized controlled trial. Front Neurol. (2023) 14:1167957. doi: 10.3389/fneur.2023.1167957 37188307 PMC10176447

[9] Chen W Li M Li H Lin Y Feng Z. Tai Chi for fall prevention and balance improvement in older adults: a systematic review and meta-analysis of randomized controlled trials. Front Public Health. (2023) 11:1236050. doi: 10.3389/fpubh.2023.1236050 37736087 PMC10509476

[10] Strain T Fitzsimons C Kelly P Mutrie N. The forgotten guidelines: cross-sectional analysis of participation in muscle strengthening and balance & co-ordination activities by adults and older adults in Scotland. BMC Public Health. (2016) 16:1108. doi: 10.1186/s12889-016-3774-6 27769211 PMC5073826

[11] De Biase S Cook L Skelton DA Witham M Ten Hove R. The COVID-19 rehabilitation pandemic. Age Ageing. (2020) 49:696–700. doi: 10.1093/ageing/afaa118 32470131 PMC7314277

[12] Di Pumpo M Miatton A Riccardi MT Graps EA Baldo V Buja A . Digital health interventions to promote physical activity in community-dwelling older adults: a systematic review and semiquantitative analysis. Int J Public Health. (2025) 69:1607720. doi: 10.3389/ijph.2024.1607720 39830161 PMC11738617

[13] Yao J Wang H Jia S Chen W Hu E. Effects of mobile health technology on physical activity in pregnant women: a systematic review and meta-analysis. BMC Pregnancy Childbirth. (2025) 25:1360. doi: 10.1186/s12884-025-08455-6 41272547 PMC12751654

[14] Zangger G Bricca A Liaghat B Juhl CB Mortensen SR Andersen RM . Benefits and harms of digital health interventions promoting physical activity in people with chronic conditions: systematic review and meta-analysis. J Med Internet Res. (2023) 25:e46439. doi: 10.2196/46439 37410534 PMC10359919

[15] Marcolino MS Oliveira JAQ D'Agostino M Ribeiro AL Alkmim MBM Novillo-Ortiz D. The impact of mHealth interventions: systematic review of systematic reviews. JMIR Mhealth Uhealth. (2018) 6:e23. doi: 10.2196/mhealth.8873 29343463 PMC5792697

[16] Li Y Wang Q Ren Y Mao X. Effects of Tai Chi based on information and communication technology for patients with mild cognitive impairment on cognitive and physical function: a systematic review and meta-analysis. Front Public Health. (2025) 12:1495645. doi: 10.3389/fpubh.2024.1495645 39839400 PMC11748305

[17] Tu Y Lin X Zhang J Guan Y Zhao B. Effects of digitalized traditional Chinese exercises on the physical and mental health and quality of life of older adults: a systematic review and meta-analysis of randomized controlled trials. Front Public Health. (2025) 13:1725847. doi: 10.3389/fpubh.2025.1725847 41458421 PMC12738345

[18] Li ML Kor PP Sui YF Liu JY. Health maintenance through home-based interventions for community-dwelling older people with sarcopenia during and after the COVID-19 pandemic: a systematic review and meta-analysis. Exp Gerontol. (2023) 174:112128. doi: 10.1016/j.exger.2023.112128 36804363 PMC9941010

[19] Wan R Huang J Wang K Long D Tao A Huang J . Effectiveness of mind-body exercise in older adults with sarcopenia and frailty: a systematic review and meta-analysis. J Cachexia Sarcopenia Muscle. (2025) 16:e13806. doi: 10.1002/jcsm.13806 40254030 PMC12009637

[20] Page MJ McKenzie JE Bossuyt PM Boutron I Hoffmann TC Mulrow CD . The PRISMA 2020 statement: an updated guideline for reporting systematic reviews. BMJ. (2021) 372:n71. doi: 10.1136/bmj.n71 33782057 PMC8005924

[21] Luo D Wan X Liu J Tong T. Optimally estimating the sample mean from the sample size, median, mid-range, and/or mid-quartile range. Stat Methods Med Res. (2018) 27:1785–805. doi: 10.1177/0962280216669183 27683581

[22] Wan X Wang W Liu J Tong T. Estimating the sample mean and standard deviation from the sample size, median, range and/or interquartile range. BMC Med Res Methodol. (2014) 14:135. doi: 10.1186/1471-2288-14-135 25524443 PMC4383202

[23] Sterne JAC Savović J Page MJ Elbers RG Blencowe NS Boutron I . RoB 2: a revised tool for assessing risk of bias in randomised trials. BMJ. (2019) 366:l4898. doi: 10.1136/bmj.l4898 31462531

[24] Guyatt G Oxman AD Akl EA Kunz R Vist G Brozek J . GRADE guidelines: 1. Introduction-GRADE evidence profiles and summary of findings tables. J Clin Epidemiol. (2011) 64:383–94. doi: 10.1016/j.jclinepi.2010.04.026 21195583

[25] GRADEpro GDT: GRADEpro Guideline Development Tool [Software]. Hamilton, ON: Evidence Prime, Inc. (2020).

[26] Chen W Yao J Wang H Jia S Zhu X Mao L. Effects of digital health interventions on muscle mass, muscle strength, and physical function in older adults with sarcopenia: a systematic review and meta-analysis. Front Public Health. (2025) 13:1711514. doi: 10.3389/fpubh.2025.1711514 41383324 PMC12689566

[27] Yao J Wang H Jia S Liao M Chen W. Effects of plaza dancing on body composition and cardiopulmonary function in middle-aged and the aged healthy women: a systematic review and meta-analysis. Front Public Health. (2025) 13:1667818. doi: 10.3389/fpubh.2025.1667818 40988663 PMC12450655

[28] Wu G Keyes L Callas P Ren X Bookchin B. Comparison of telecommunication, community, and home-based Tai Chi exercise programs on compliance and effectiveness in elders at risk for falls. Arch Phys Med Rehabil. (2010) 91:849–56. doi: 10.1016/j.apmr.2010.01.024 20510973

[29] Li X Zou L Li H. Tai Chi movement recognition and precise intervention for the elderly based on inertial measurement units and temporal convolutional neural networks. Sensors. (2024) 24:4208. doi: 10.3390/s24134208 39000985 PMC11244047

[30] Hsieh CC Lin PS Hsu WC Wang JS Huang YC Lim AY . The effectiveness of a virtual reality-based Tai Chi exercise on cognitive and physical function in older adults with cognitive impairment. Dement Geriatr Cogn Disord. (2018) 46:358–70. doi: 10.1159/000494659 30537752

[31] Li F Harmer P Eckstrom E Fitzgerald K Winters-Stone K. Clinical effectiveness of cognitively enhanced Tai Ji Quan training on global cognition and dual-task performance during walking in older adults with mild cognitive impairment or self-reported memory concerns: a randomized controlled trial. Ann Intern Med. (2023) 176:1498–507. doi: 10.7326/M23-1603 37903365 PMC13527859

[32] Zhang Y Li H Huang R. The effect of Tai Chi (Bafa Wubu) training and artificial intelligence-based movement-precision feedback on the mental and physical outcomes of elderly. Sensors. (2024) 24:6485. doi: 10.3390/s24196485 39409525 PMC11479303

[33] He S Meng D Wei M Guo H Yang G Wang Z. Proposal and validation of a new approach in tele-rehabilitation with 3D human posture estimation: a randomized controlled trial in older individuals with sarcopenia. BMC Geriatr. (2024) 24:586. doi: 10.1186/s12877-024-05188-7 38977995 PMC11232209

[34] Chen PJ Penn IW Wei SH Chuang LR Sung WH. Augmented reality-assisted training with selected Tai-Chi movements improves balance control and increases lower limb muscle strength in older adults: a prospective randomized trial. J Exerc Sci Fit. (2020) 18:142–7. doi: 10.1016/j.jesf.2020.05.003 32514277 PMC7265060

[35] Pillay J Gaudet LA Saba S Vandermeer B Ashiq AR Wingert A . Falls prevention interventions for community-dwelling older adults: systematic review and meta-analysis of benefits, harms, and patient values and preferences. Syst Rev. (2024) 13:289. doi: 10.1186/s13643-024-02681-3 39593159 PMC11590344

[36] Lin J Ning S Lyu S Gao H Shao X Tan Z . The effects of different types of Tai Chi exercises on preventing falls in older adults: a systematic review and network meta-analysis. Aging Clin Exp Res. (2024) 36:65. doi: 10.1007/s40520-023-02674-7 38472538 PMC10933200

[37] Collado-Mateo D Lavín-Pérez AM Peñacoba C Del Coso J Leyton-Román M Luque-Casado A . Key factors associated with adherence to physical exercise in patients with chronic diseases and older adults: an umbrella review. Int J Environ Res Public Health. (2021) 18:2023. doi: 10.3390/ijerph18042023 33669679 PMC7922504

[38] Shi Y Stanmore E McGarrigle L Todd C. Effectiveness of digital health exercise interventions on muscle function and physical performance in older adults with possible, confirmed or severe sarcopenia: a protocol for a systematic review. BMJ Open. (2024) 14:e086124. doi: 10.1136/bmjopen-2024-086124 39433420 PMC11499845

[39] Kümmel J Kramer A Giboin LS Gruber M. Specificity of balance training in healthy individuals: a systematic review and meta-analysis. Sports Med. (2016) 46:1261–71. doi: 10.1007/s40279-016-0515-z 26993132

[40] Schoenfeld BJ. The mechanisms of muscle hypertrophy and their application to resistance training. J Strength Cond Res. (2010) 24:2857–72. doi: 10.1519/JSC.0b013e3181e840f3 20847704

[41] Xiang W Wang JY Ji BJ Li LJ Xiang H. Effectiveness of different telerehabilitation strategies on pain and physical function in patients with knee osteoarthritis: systematic review and meta-analysis. J Med Internet Res. (2023) 25:e40735. doi: 10.2196/40735 37982411 PMC10728785

[42] Gómez-Redondo P Valenzuela PL Morales JS Ara I Mañas A. Supervised versus unsupervised exercise for the improvement of physical function and well-being outcomes in older adults: a systematic review and meta-analysis of randomized controlled trials. Sports Med. (2024) 54:1877–906. doi: 10.1007/s40279-024-02024-1 38647999 PMC11258164

[43] Costa SN Ferreira LHB Bento PCB. Effects of home-based exercise programs on mobility, muscle strength, balance, and gait in community-dwelling older adults: a systematic review and meta-analysis. J Aging Phys Act. (2023) 31:693–704. doi: 10.1123/japa.2022-0221 36623512

