# Supplementary material for: Effects of digitalized Tai Chi intervention on muscle function and physical performance in older adults: a systematic review and meta-analysis
Source: Front Public Health. 2026 Mar 18;14:1793728. doi: 10.3389/fpubh.2026.1793728 (PMC13038443; doi:10.3389/fpubh.2026.1793728)
Supplement: Supplementary file 1 [file Supplementary_file_1.docx]

**Supplementary Text 1 Search Strategies**

1. **PubMed：**

search strategy：

#1 "Aged"[MeSH Terms]

#2 "older adults"[Title/Abstract] OR "elderly"[Title/Abstract] OR "Aged"[Title/Abstract]

#3 #1 OR #2

#4 "Digital Technology"[MeSH Terms] OR "Telemedicine"[MeSH Terms] OR "Virtual Reality"[MeSH Terms] OR "Artificial Intelligence"[MeSH Terms]

#5 "digital"[Title/Abstract] OR "tele*"[Title/Abstract] OR "online"[Title/Abstract] OR "Virtual Reality"[Title/Abstract] OR "VR"[Title/Abstract] OR "augmented reality"[Title/Abstract] OR "AR"[Title/Abstract] OR "exergame"[Title/Abstract] OR "sensor"[Title/Abstract] OR "videoconferencing"[Title/Abstract] OR "pose estimation"[Title/Abstract]

#6 #4 OR #5

#7 "Tai Ji"[MeSH Terms]

#8 "Tai Chi"[Title/Abstract] OR "Tai Ji Quan"[Title/Abstract] OR "Taiji"[Title/Abstract]

#9 #7 OR #8

#10 "Randomized Controlled Trial"[Publication Type]

#11 "randomized"[Title/Abstract] OR "randomised"[Title/Abstract] OR "controlled trial"[Title/Abstract] OR "randomly"[Title/Abstract] OR "RCT"[Title/Abstract]

#12 #10 OR #11

#13 #3 AND #6 AND #9 AND #12

1. **Web of Science：**

search strategy:

#1 TS=("aged" OR "older adult*" OR "elderly" OR "senior*" OR "geriatric*")

#2 TS=("Digital Technology" OR "Telemedicine" OR "Virtual Reality" OR "Artificial Intelligence" OR "Mobile Application*")

#3 TS=("digital" OR "tele*" OR "online" OR "virtual reality" OR "VR" OR "augmented reality" OR "AR" OR "exergame" OR "sensor*" OR "videoconferencing" OR "pose estimation")

#4 #2 OR #3

#5 TS=("Tai Ji" OR "Tai Chi")

#6 TS=("Tai Ji Quan" OR "Taiji")

#7 #5 OR #6

#8 TS=("randomized controlled trial" OR "randomized" OR "randomised" OR "controlled trial" OR "randomly" OR "RCT")

#9 #1 AND #4 AND #7 AND #8

1. **Embase：**

search strategy:

#1 'aged'/exp OR 'chronic disease'/exp

#2 'older adult*':ti,ab OR 'elderly':ti,ab OR 'senior*':ti,ab OR 'geriatric*':ti,ab

#3 #1 OR #2

#4 'telemedicine'/exp OR 'digital health'/exp OR 'virtual reality'/exp OR 'artificial intelligence'/exp OR 'mobile application'/exp

#5 'digital':ti,ab OR 'tele*':ti,ab OR 'online':ti,ab OR 'virtual reality':ti,ab OR 'vr':ti,ab OR 'augmented reality':ti,ab OR 'ar':ti,ab OR 'exergame':ti,ab OR 'sensor*':ti,ab OR 'videoconferencing':ti,ab OR 'pose estimation':ti,ab

#6 #4 OR #5

#7 'tai chi'/exp

#8 'tai chi':ti,ab OR 'tai ji':ti,ab OR 'tai ji quan':ti,ab OR 'taiji':ti,ab

#9 #7 OR #8

#10 'randomized controlled trial'/exp OR 'randomization'/exp OR 'placebo'/exp

#11 'randomized':ti,ab OR 'randomised':ti,ab OR 'controlled trial':ti,ab OR 'randomly':ti,ab OR 'rct':ti,ab

#12 #10 OR #11

#13 #3 AND #6 AND #9 AND #12

1. **Cochrane Library：**

search strategy:

#1 [mh "Aged"]

#2 "older adult*" OR "elderly" OR "senior*" OR "geriatric*":ti,ab,kw

#3 [mh "Chronic Disease"]

#4 "chronic disease*":ti,ab,kw

#5 #1 OR #2 OR #3 OR #4

#6 [mh "Digital Technology"] OR [mh "Telemedicine"] OR [mh "Virtual Reality"] OR [mh "Artificial Intelligence"]

#7 "digital" OR "tele*" OR "online" OR "virtual reality" OR "VR" OR "augmented reality" OR "AR" OR "exergame" OR "sensor*" OR "videoconferencing" OR "pose estimation":ti,ab,kw

#8 #6 OR #7

#9 [mh "Tai Ji"]

#10 "Tai Chi" OR "Tai Ji Quan" OR "Taiji":ti,ab,kw

#11 #9 OR #10

#12 [mh "Randomized Controlled Trial"] OR [mh "Placebos"]

#13 "randomized" OR "randomised" OR "controlled trial" OR "randomly" OR "RCT" OR "placebo":ti,ab,kw

#14 #12 OR #13

#15 #5 AND #8 AND #11 AND #14

1. **CINAHL：**

search strategy:

#1 (MH "Aged")

#2 TX ( "older adult*" OR elderly OR senior* OR geriatric* )

#3 #1 OR #2

#4 (MH "Telemedicine+") OR (MH "Virtual Reality") OR (MH "Artificial Intelligence") OR (MH "Mobile Applications")

#5 TX ( "digital" OR tele* OR online OR "virtual reality" OR VR OR "augmented reality" OR AR OR exergame OR sensor* OR "videoconferencing" OR "pose estimation" )

#6 #4 OR #5

#7 (MH "Tai Chi")

#8 TX ( "Tai Chi" OR "Tai Ji" OR "Tai Ji Quan" OR Taiji )

#9 #7 OR #8

#10 (MH "Randomized Controlled Trials")

#11 TX ( randomized OR randomised OR "controlled trial" OR randomly OR RCT )

#12 #10 OR #11

#13 #3 AND #6 AND #9 AND #12

1. **CNKI：**

search strategy:

#1 老年人+高龄+社区老人

#2 太极+太极拳

#3 数字化+虚拟现实+远程

#4 随机+随机对照实验

#5 #1 AND #2 AND #3 AND #4

1. **VIP：**

search strategy:

#1 主题:("老年人" OR "高龄" OR "社区老人" OR "老人")

#2 主题:("太极拳" OR "太极")

#3 主题:("数字化" OR "远程" OR "虚拟现实" OR "VR" OR "增强现实" OR "AR" OR "人工智能" OR "AI" OR "可穿戴" OR "传感器" OR "智能手机" OR "App" OR "姿态识别" OR "视频会议")

#4 主题:("随机" OR "随机对照" OR "RCT" OR "临床试验")

#5 #1 AND #2 AND #3 AND #4

1. **Wangfang：**

search strategy:

#1 (M=老年人 OR M=高龄 OR M=社区老人 OR M=老人)

#2 (M=太极拳 OR M=太极)

#3 (M=数字化 OR M=远程 OR M=虚拟现实 OR M=VR OR M=增强现实 OR M=AR OR M=人工智能 OR M=AI OR M=可穿戴 OR M=传感器 OR M=智能手机 OR M=App OR M=姿态识别 OR M=视频会议)

#4 (M=随机 OR M=随机对照 OR M=RCT OR M=临床试验)

#5 #1 AND #2 AND #3 AND #4
