# Supplementary material for: Effects of digitalized Tai Chi intervention on muscle function and physical performance in older adults: a systematic review and meta-analysis
Source: Front Public Health. 2026 Mar 18;14:1793728. doi: 10.3389/fpubh.2026.1793728 (PMC13038443; doi:10.3389/fpubh.2026.1793728)
Supplement: Supplementary file 2 [file Data_Sheet_1.docx]

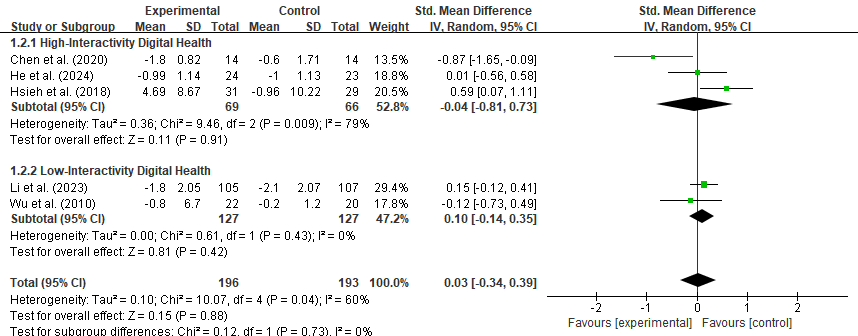


Supplementary Figure 1 Forest plot for the subgroup analysis of technological interaction levels on the effects of digitalized Tai Chi interventions on the Timed Up and Go Test in older adults


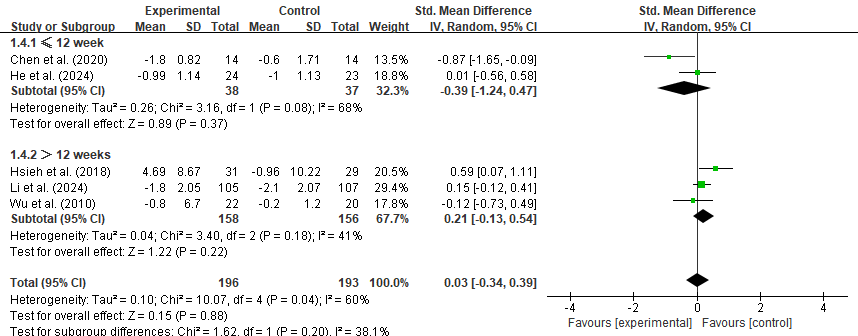


Supplementary Figure 2 Forest plot for the subgroup analysis of intervention duration on the effects of digitalized Tai Chi interventions on the Timed Up and Go Test in older adults
